# Auditory Cortex Basal Activity Modulates Cochlear Responses in Chinchillas

**DOI:** 10.1371/journal.pone.0036203

**Published:** 2012-04-30

**Authors:** Alex León, Diego Elgueda, María A. Silva, Carlos M. Hamamé, Paul H. Delano

**Affiliations:** 1 Laboratorio de Neurobiología de la Audición, Programa de Fisiología y Biofísica, ICBM, Facultad de Medicina, Universidad de Chile, Santiago, Chile; 2 Institute of Systems Research and Neuroscience and Cognitive Sciences Program, University of Maryland, College Park, Maryland, United States of America; 3 Lyon Neuroscience Research Center (INSERM U1028 - CNRS UMR5292), Brain Dynamics and Cognition Team, Lyon, France; 4 Servicio Otorrinolaringología, Hospital Clínico de la Universidad de Chile, Santiago, Chile; Hotchkiss Brain Institute, University of Calgary, Canada

## Abstract

**Background:**

The auditory efferent system has unique neuroanatomical pathways that connect the cerebral cortex with sensory receptor cells. Pyramidal neurons located in layers V and VI of the primary auditory cortex constitute descending projections to the thalamus, inferior colliculus, and even directly to the superior olivary complex and to the cochlear nucleus. Efferent pathways are connected to the cochlear receptor by the olivocochlear system, which innervates outer hair cells and auditory nerve fibers. The functional role of the cortico-olivocochlear efferent system remains debated. We hypothesized that auditory cortex basal activity modulates cochlear and auditory-nerve afferent responses through the efferent system.

**Methodology/Principal Findings:**

Cochlear microphonics (CM), auditory-nerve compound action potentials (CAP) and auditory cortex evoked potentials (ACEP) were recorded in twenty anesthetized chinchillas, before, during and after auditory cortex deactivation by two methods: lidocaine microinjections or cortical cooling with cryoloops. Auditory cortex deactivation induced a transient reduction in ACEP amplitudes in fifteen animals (deactivation experiments) and a permanent reduction in five chinchillas (lesion experiments). We found significant changes in the amplitude of CM in both types of experiments, being the most common effect a CM decrease found in fifteen animals. Concomitantly to CM amplitude changes, we found CAP increases in seven chinchillas and CAP reductions in thirteen animals. Although ACEP amplitudes were completely recovered after ninety minutes in deactivation experiments, only partial recovery was observed in the magnitudes of cochlear responses.

**Conclusions/Significance:**

These results show that blocking ongoing auditory cortex activity modulates CM and CAP responses, demonstrating that cortico-olivocochlear circuits regulate auditory nerve and cochlear responses through a basal efferent tone. The diversity of the obtained effects suggests that there are at least two functional pathways from the auditory cortex to the cochlea.

## Introduction

Sensory systems are usually thought as biological receptors that transduce external energy into bioelectrical signals, which are conducted through ascending pathways from sensory epithelia to the brain. However, it is known that auditory, vestibular and muscle spindle receptors have conspicuous descending projections that connect the brain with receptor cells [1]–[4]. These efferent systems form feedback loops that could modulate afferent responses even at the level of sensory transduction.

The corticofugal descending auditory system is a complex neuronal network that comprises the primary auditory cortex, thalamus, inferior colliculus (IC), cochlear nucleus (CN), superior olivary complex, and reaches cochlear receptor cells by olivocochlear fibers [5]–[7]. According to their anatomic origin, olivocochlear neurons are classified into the medial (MOC) and lateral (LOC) olivocochlear systems [8]. The majority of MOC fibers cross to the contralateral cochlea and contact outer hair cells (OHC) by cholinergic synapses mediated via exclusive nicotinic receptors constituted of alpha-9/alpha-10 subunits [9], [10]. In contrast, most LOC fibers make axo-dendritic synapses with ipsilateral auditory-nerve afferent fibers and release a variety of neurotransmitters like: acetylcholine, dopamine and neuropeptides [11].

The physiology of auditory efferents has been studied by electrical stimulation, or synaptic transmission manipulation at different levels of the efferent pathway. For instance, electrical activation of MOC fibers reduces the amplitude of the auditory-nerve compound action potentials (CAP) and increases cochlear microphonics (CM) receptor potentials [12]–[15], while blockade of dopamine cochlear receptors or selective lesions of LOC fibers produce CAP reductions without affecting CM potentials or otoacoustic emissions (measures of OHC function) [16]–[18]. Besides, galvanic stimulation of the IC can produce either reductions or enhancements in CAP amplitudes, accompanied by CM increases [19] or by no CM amplitude changes [20].

Electrical microstimulation of the auditory cortex facilitates or inhibits contralateral and ipsilateral CN responses in mice, depending on whether there is a match between the cortical and CN characteristic frequencies [21], [22]. Moreover, auditory-cortex microstimulation also modulates CM amplitudes in mustached bats, producing decreases or enhancements depending on stimulus frequency, and the side of the cortical stimulation [23], [24]. In humans, electrical stimulation of the contralateral auditory cortex in patients with refractory epilepsy reduced the amplitude of evoked otoacoustic emissions while there was no change under stimulation of non-auditory cortical areas [25].

Corticofugal modulation of peripheral auditory responses has also been evaluated by ablation studies in humans [26] and pharmacological auditory cortex deactivation in bats [23], [24], [27], [28]. For example, surgical resection of the temporal lobe in epileptic patients produced a reduction in MOC activity, demonstrating that the temporal lobe modulates the amplitude of otoacoustic emissions recorded from the contralateral ear [26]. Muscimol auditory cortex deactivation in mustached bats produced a reduction of CM amplitude at 61 kHz [23], while bicuculline -an antagonist of GABA-A receptors- reversed corticofugal effects of microstimulation [24]. Altogether, these results strongly suggest that the efferent pathway from the auditory cortex to the cochlea is functional, providing a top-down regulation of OHC function, however its role in auditory nerve activity is still under assessment [20], [29], [30].

In awake animals, a putative function for MOC fibers is the modulation of cochlear sensitivity by means of an efferent basal tone. Indeed, Zheng and collaborators found CM amplitude reductions after sectioning the olivocochlear bundle in chinchillas suggesting the presence of an inhibitory basal MOC tone that regulates cochlear afferent responses [31]. However, it is not clear whether this tone is restricted to the olivocochlear system or if it comprises corticofugal descending pathways. We propose that auditory-cortex basal activity modulates cochlear sensitivity through the cortico-olivocochlear efferent system. Here, we recorded CM and CAP responses in anesthetized chinchillas before, during and after auditory cortex deactivation using two methods: (i) lidocaine microinjections [32] and (ii) transdural cooling with cryoloops [33]. We found that both cortical deactivation methods produced significant CAP and CM amplitude changes, demonstrating that auditory-cortex basal activity exerts tonic influences on auditory nerve and cochlear responses.

## Materials and Methods

### Ethics statement

All procedures involving animals were made in accordance with NIH Guidelines for the Care and Use of Laboratory Animals, publication No. 86-23, revised 1996, and were approved by the Institutional Bioethics Committee (Comité de Bioética de Investigación en Animales, Facultad de Medicina, Universidad de Chile, permit number CBA #0262). All surgery was performed under ketamine and xylazine anesthesia, and every effort was made to minimize suffering.

### Subjects

Twenty adult chinchillas (*Chinchilla laniger*) weighing 400 to 700 g were anesthetized with ketamine (30 mg/Kg), xylazine (4 mg/Kg) and atropine (0.04 mg/kg, I.M.) and were maintained under anesthesia with repeated half doses every 30–45 minutes, or when necessary, judging by the foot-withdrawal reflex. Rectal temperature was maintained at 35–37°C by means of a heating pad. At the end of each experiment, deeply anesthetized animals were humanely euthanized with an overdose of sodium thiopental (120 mg/kg).

### Surgical procedures

*(i)* Cochlear surgery: in all animals, the right pinna was resected for proper access to the external auditory meatus and tympanic membrane. A dorsal opening was made in the bulla to allow cutting of the tensor tympani muscle. The right cochlea was accessed by a posterior aperture of the tympanic bulla, and a silver electrode (80 µm) was positioned in the round window. This approach allowed us to detach the stapedius muscle from its insertion in all animals used. In two animals, we also performed a left cochlear surgery, and silver round window electrodes were positioned bilaterally. *(ii)* Auditory-cortex surgery: an extended craniotomy was performed in the temporal bone to expose the left auditory cortex of the chinchillas following descriptions given by Harrison and collaborators [34], [35]. The dura mater was incised, and a nichrome electrode (200 µm) was positioned and lowered (500 µm) into the left auditory cortex using a stereotaxic arm (Stoelting®) and a hydraulic micro-drive (David Kopf® Instruments, model 1207-B).

### Auditory stimuli

Tones (1–8 kHz) and clicks (100 µs wide) were digitally generated by a National Instruments® PCI board (6071-E) at 100,000 samples/s, attenuated by a PA-5 programmable attenuator and delivered with an EC1 electrostatic speaker (Tucker Davis Technologies® system III). Tone duration was 15 ms with a 5 ms rise/fall time. Both types of stimuli were delivered at a rate of 4 Hz and at intensities of 20 to 100 dB SPL, through tubes sealed to the external auditory meatus with ear impression material (Innovation Meditech Gmbh®). During experiments, sound pressure levels were evaluated in a sequential order, from low to high intensity levels, except experiment cx_rw_01, in which only a single sound pressure level was evaluated. Calibrations were performed with a Knowles microphone.

### Electrophysiological recordings

Auditory cortex evoked potentials (ACEP) were always recorded from the left hemisphere and cochlear responses from the right round window. In two animals, we also recorded cochlear potentials from the left round window. The round window signal was amplified 10,000×, and filtered between 300–10,000 and 300–20,000 Hz for low and high-frequency tones respectively, using a BMA-200 amplifier (Cwe-inc®). A low impedance (<5 kΩ) Nichrome® electrode (200 µm) was positioned in the left auditory cortex. The signal was amplified 10,000× and band-pass filtered (1–1000 Hz) using a BMA-400 amplifier (Cwe-inc®) and a low-pass filtered at 200 Hz (Frequency devices® 901). Both signals were acquired and digitized at 40,000 samples/s with a National Instruments® multifunction board (6071-E). Experiments were controlled with custom made programs developed in C language (LabWindows CVI® environment).

### Lidocaine cortical deactivation (n = 10)

A Hamilton microsyringe (10 µl) was positioned next to the cortical electrode in the left auditory cortex. A 3 µl lidocaine (2%) or saline microinjection was given at a rate of 1 µl/min (500 µm depth). According to Tehovnik and Sommer [32], the radio of diffusion of a cortical lidocaine microinjection follows a volume distribution, where volume (µl) = 4/3*π*r^3^, predicting that a 3 µl lidocaine microinjection will spread around 0.9 mm in the cerebral cortex. The penetration site was completely covered with agar. The Hamilton microsyringe was controlled using a manual stereotaxic injector (Stoelting®). An input-output curve of click-evoked cortical potentials was used to measure and control auditory cortex deactivation.

### Cryoloop cortical deactivation (n = 5)

A custom made cryoloop [33] was positioned in the left auditory cortex, and a circuit of cold methanol was used to decrease cortical temperature in a range between 2° and 8°C. Two animals were tested at two temperatures: the first at 8° and 4°C; and the second at 4° and 2°C. The other three chinchillas were tested at a single temperature each: 8°, 3°, and 2°C. Loop sizes positioned in the cortical surface were about 3–4 mm. Cortical temperature was measured in each recording (every one to two minutes) using a fine thermocouple (Cole-Parmer®) attached to cryoloops. After turning off the cryoloop pump, there was a passive warming (recovery period) of the auditory cortex related to the cerebral blood flow. In two experiments, we simultaneously recorded cortical and cochlear temperature with an additional thermocouple positioned at the promontory of the tympanic cavity.

### Auditory cortex lesion (n = 5)

A third experimental group in which there was no recovery in ACEP amplitude after cortical deactivation (four with lidocaine and one with a cryoloop), was included in the analysis as a separate group (lesion group, see Table 1).

**Table 1:** Summary of results.

Side of recording			Right cochlea		Left cochlea		Left auditory cortex
n	Exp_ID	Method	CAP (dB)	CM (dB)	CAP (dB)	CM (dB)	ACEP (dB)
**1**	**cx_rw_01**	**L**	−2.6	−1.2			−8.4
**2**	**cx_rw_02**	**L**	5.3	−2.4			−12.1
**3**	**cx_rw_06**	**L**	−9.5	−7.5			−21.4
**4**	**cx_rw_07**	**L**	−3.5	−3.2			−9.5
**5**	**cx_rw_08**	**L**	4.4	2.0			−12.7
**6**	**cx_rw_09**	**L**	−4.5	3.0			−14.7
**7**	**cx_rw_12**	**L**	−3.6	−3.3	−1.4	−1.9	−14.5
**8**	**cx_rw_13**	**L**	−4.5	−2.7			−7.5
**9**	**cx_rw_14**	**L**	3.7	3.0			−11.4
**10**	**cx_rw_19**	**L**	−2.0	−3.3	1.5	3.5	−6.2
**11**	**cx_rw_17**	**2°C**	−2.7	−1.5			−25.5
**12**	**cx_rw_22**	**3°C**	−4.0	−3.3			−27.7
**13**	**cx_rw_23**	**4-2°C**	1.1	−1.8			−17.6
**14**	**cx_rw_30**	**8°C**	−5.0	−2.7			−16.2
**15**	**cx_rw_31**	**8-4°C**	−8.3	−4.3			−25.2
**16**	**lesion_1**	**L**	2.8	−8.2			−10.7
**17**	**lesion_2**	**L**	1.7	1.5			−11.8
**18**	**lesion_3**	**L**	−10.1	−12.0			−7.6
**19**	**lesion_4**	**L**	2.3	3.6			−17.2
**20**	**lesion_5**	**2°C**	−1.5	−0.5			−26.5

The largest changes in CAP, CM and ACEP measured in dB for each experiment. Deactivation experiments included fifteen animals (n = 1–15) but seventeen ears, as there were two bilateral experiments. Five chinchillas were tested in lesion experiments (n = 16–20). Exp_ID: Identification of experiments. L: lidocaine experiments. C°: cortical temperature of cryoloop experiments.

### Data analysis

**Figure 1:**
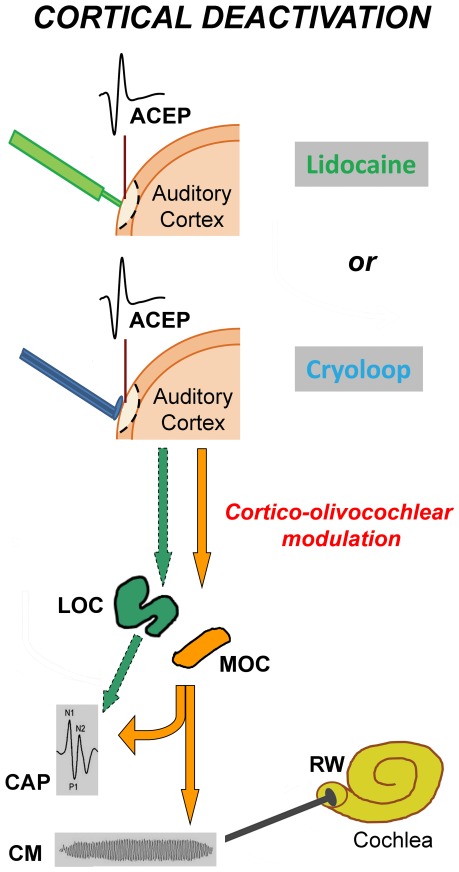
Schematic illustration of experimental procedures. In all deactivation experiments (n = 15), auditory cortex evoked potentials (ACEP) were recorded from the left auditory cortex, and cochlear potentials (CAP and CM) from the right round window (RW) (contralateral experiments), while in two cases, left cochlear potentials were also obtained (bilateral experiments). Two methods were used in separate experiments to deactivate the auditory cortex: (i) cortical microinjection of lidocaine (n = 10) and (ii) cortical cooling with cryoloops (n = 5). Cortico-olivocochlear effects of auditory cortex deactivation were evaluated by measuring cochlear and auditory neural responses (CM and CAP) before and after cortical deactivation. We propose the presence of two descending pathways from the auditory cortex to the inferior colliculus (not shown in this figure), and from IC to medial and lateral olivocochlear neurons, represented in orange (MOC) and green (LOC) colors respectively. A corticofugal modulation of MOC activity (orange colored arrows) would modify both CAP and CM responses, while a cortical modulation of LOC activity (green colored arrows) would only affect CAP responses.

In each experiment, cochlear and cortical potentials in response to auditory stimuli at different frequencies and sound pressure levels were evaluated before, and after auditory cortex deactivation (Figures 1 and S1). The averages and standard deviations from 48–64 trials were used to calculate the amplitudes of the cochlear and cortical potentials. The significance of the differences between evoked cortical and cochlear responses during the control period and during cortical deactivation were determined in lidocaine experiments using t-tests, while repeated measures ANOVA and Tukey post-hoc tests were used in the cryoloop experiments (alpha = 0.05). The amplitudes of CAP, CM, and auditory-cortex evoked potentials were referenced in dB to the amplitude obtained at a given frequency and sound pressure level prior to cortical deactivation [amplitude change (dB) = 20*LOG_10_ (amplitude/reference amplitude)].

#### Cochlear potentials

Acoustic stimuli were presented with alternating polarity in order to allow us to separate CAPs from CMs. Subtraction of the averaged responses to stimuli of the two polarities allowed computation of the amplitudes of CMs by performing a Fast Fourier Transform (FFT) in a 12.8 ms window after the CAP response (using a custom-made C program, LabWindows®), while the amplitudes of the compound action potential of the auditory nerve were measured as the difference between the N1 and P1 peaks.

#### Cortical potentials

ACEP amplitudes were calculated by measuring the amplitude of the averaged response using a time window from 5 to 50 ms after the onset of the auditory stimulus.

## Results

Lidocaine microinjections and cryoloops produced ACEP amplitude reductions in all studied chinchillas (n = 20, Table 1), including both lesion (n = 5) and deactivation experiments (n = 15). Unless stated, results presented in the following sections were obtained in the fifteen chinchillas in which there was a complete recovery in the amplitudes of cortical potentials (deactivation experiments).

Auditory cortex deactivation with lidocaine and cryoloops produced significant changes in the amplitude of ACEPs, CMs and CAPs in all deactivation experiments (n = 15, see figure legends for statistical values). The mean amplitude reduction in ACEP was significantly larger with cryoloop (−22.4±2.3 dB; mean ± SE) than for lidocaine (−11.8±1.4 dB) deactivation experiments (unpaired t-test, t = 4.16, p<0.01). A summary of the maximum effects obtained with both methods following cortical deactivation is shown in Table 1. To give a clear description of the lidocaine and cryoloop deactivation experiments, these results will be presented in separate sections.

### Lidocaine deactivation experiments (n = 10)

**Figure 2:**
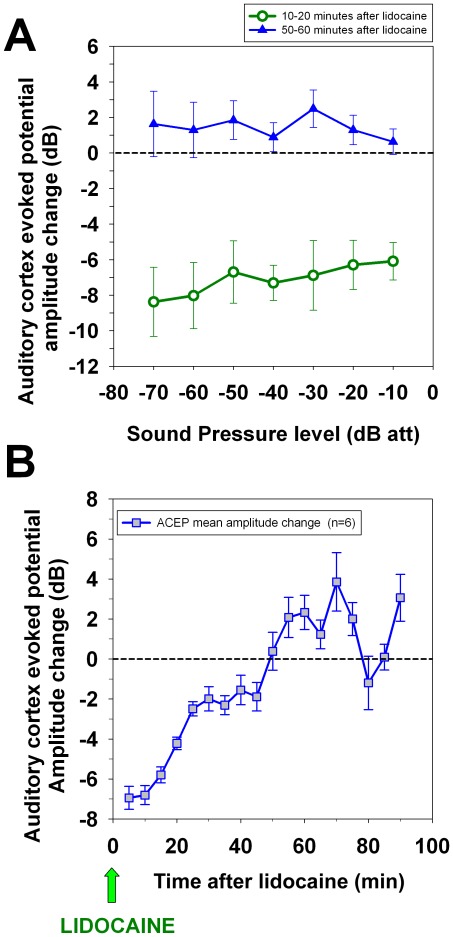
Effects of lidocaine microinjections on the amplitudes of auditory cortex evoked potentials. (A) Average input-output functions (n = 6) obtained at two different epochs after lidocaine microinjection (i) 10 to 20 and (ii) 50 to 60 minutes after the pharmacological deactivation (3 µl of 2% lidocaine at a rate of 1 µl/min). Note that ten to twenty minutes after the lidocaine microinjection a transient reduction in ACEP was observed, while fifty to sixty minutes after the microinjection a complete recovery was attained. 0 dB of attenuation corresponds approximately to 100 dB SPL. (B) Temporal course of lidocaine cortical deactivation. Mean auditory-cortex evoked potentials amplitude changes calculated from six chinchillas were measured at different epochs after a single lidocaine microinjection (each symbol represents mean ± standard error). A complete ACEP amplitude recovery was achieved 50 to 60 minutes after the lidocaine was given.

Lidocaine cortical microinjections produced reversible amplitude reductions of auditory cortex evoked potentials in all chinchillas tested (n = 10). In contrast, no significant changes were obtained after saline microinjections (n = 3, Figure S2). Cortical responses were either suppressed or reduced during the 30 to 60 minutes after the lidocaine microinjection and were completely recovered by 60 to 90 minutes after the pharmacological treatment (Figure 2).

**Figure 3:**
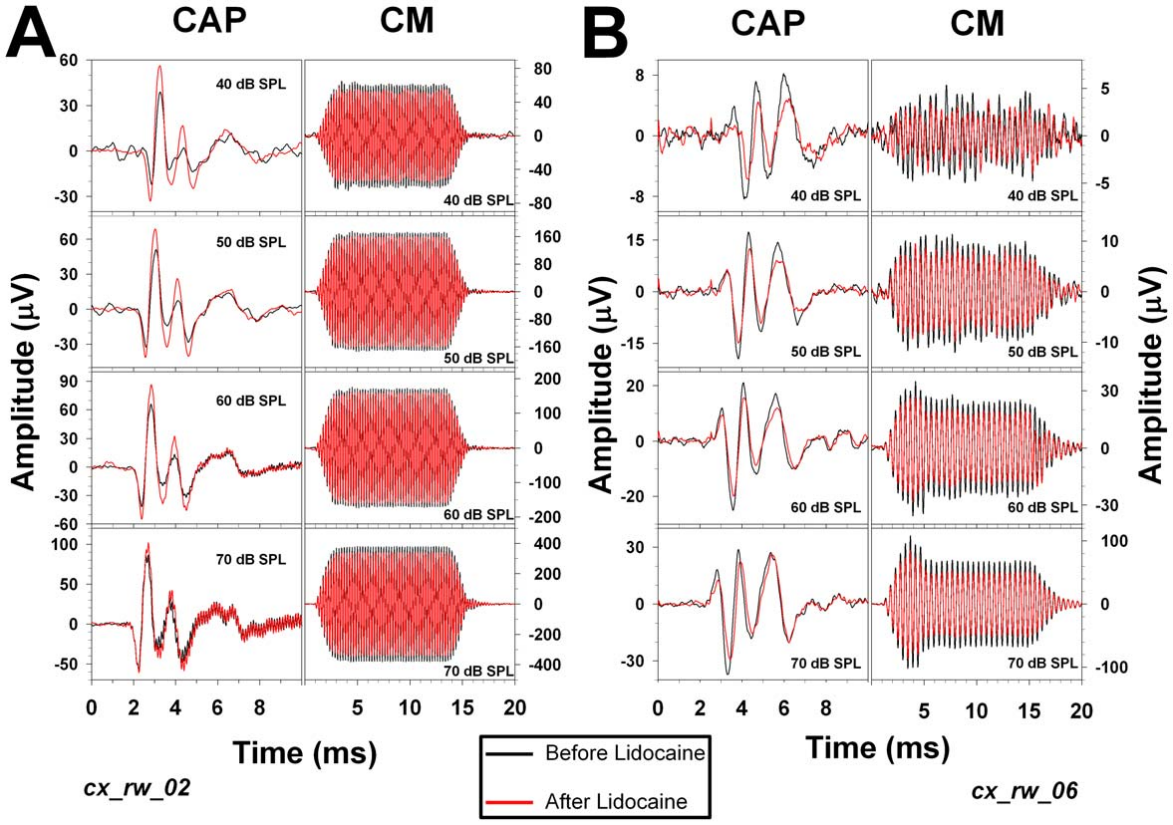
Examples of CAP and CM changes produced in the right cochlea after lidocaine microinjection in the left auditory cortex. Cochlear potentials (CAP and CM) recorded before (black traces) and after (red traces) cortical deactivation. Both examples (A and B) were obtained from different chinchillas. **A.** Significant CAP enhancements (t = −6.42, p<0.01) and CM reductions (t = 2.51, p<0.05) after cortical microinjection of lidocaine. In this example (cx_rw_02), 4 kHz stimuli were presented at different sound pressure levels. **B.** Significant CAP and CM reductions (t = 3.62, p<0.05 and t = 2.92, p<0.05 respectively) following cortical microinjection with lidocaine. In this example (cx_rw_06), 2 kHz stimuli were presented at different sound pressure levels. Note that the corticofugal effects presented in this figure are larger with low intensity stimuli and that in both experiments we obtained CM reductions, but accompanied in one case by enhancements in CAP and in the other by reductions.

**Figure 4:**
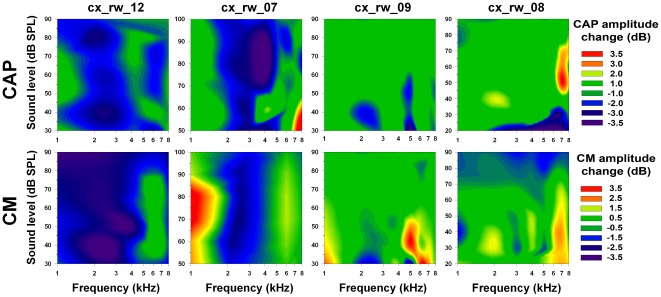
Sound pressure and frequency dependencies of CAP and CM amplitude changes after cortical deactivation with lidocaine. Each column corresponds to one experiment. The upper and lower rows display CAP and CM amplitude changes (dB) respectively. Data were acquired up to 80 minutes after the lidocaine microinjection. Green color represents no amplitude changes (±1.0 dB in CAP and ±0.5 dB in CM); blue and violet represent significant decreases; while red and yellow, significant increases. **Experiment cx_rw_12:** Significant reductions in CAP and CM (t = 2.84, p<0.05 and t = 2.81, p<0.05 respectively) were obtained at frequencies between 2–4 kHz. **Experiment cx_rw_07:** Significant CAP and CM reductions (t = 3.36, p<0.05 and t = 2.81, p<0.05 respectively) were obtained at frequencies between 2–3 kHz. **Experiment cx_rw_09:** Significant CAP reductions and CM augmentations (t = 4.34, p<0.01 and t = −2.67, p<0.05 respectively) were obtained at 5 kHz and between 5–6 kHz correspondingly. **Experiment cx_rw_08:** Significant increases in CAP and CM (t = −3.14, p<0.05 and t = −3.34, p<0.01 respectively) were obtained at frequencies of 7–8 kHz. Note that in the two left columns (Exp_ID: cx_rw_12 and cx_rw_07) CAP and CM reductions are greatest for 2–4 kHz frequencies, while small areas of either increase or decrease can be observed in the two right columns (Exp_ID: cx_rw_09 & cx_rw_08) at frequencies higher than 4 kHz.

**Figure 5:**
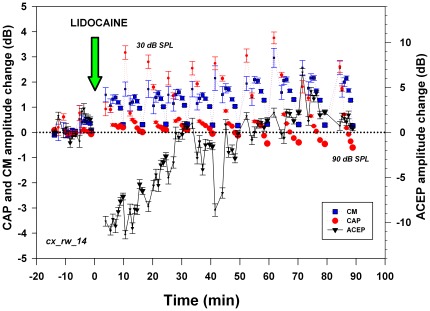
Temporal course and sound pressure dependency of amplitude changes in cochlear and cortical potentials after lidocaine cortical deactivation. Amplitude changes are depicted in dB referenced to baseline amplitude before lidocaine microinjection for CM (blue squares), CAP (red circles) and ACEP (black triangles). In this experiment, 6 kHz stimuli were presented at different sound pressure levels. Different symbol sizes represent stimulus sound pressure level, increasing in 10 dB steps from 30 to 90 dB SPL. Significant CAP and CM increases (t = −3.22, p<0.01 and t = −10.13, p<0.001 respectively) were obtained, after a 3 µl lidocaine microinjection (green arrow) in the contralateral auditory cortex. The largest effects on CM and CAP were found with low intensity sounds. Note that CM and CAP increases are maintained after the recovery of cortical evoked potentials. Note also that sixty minutes after the lidocaine microinjection, an inverted effect was observed for CAP obtained at high intensity levels (80–90 dB SPL). (Exp_ID: cx_rw_14).

**Figure 6:**
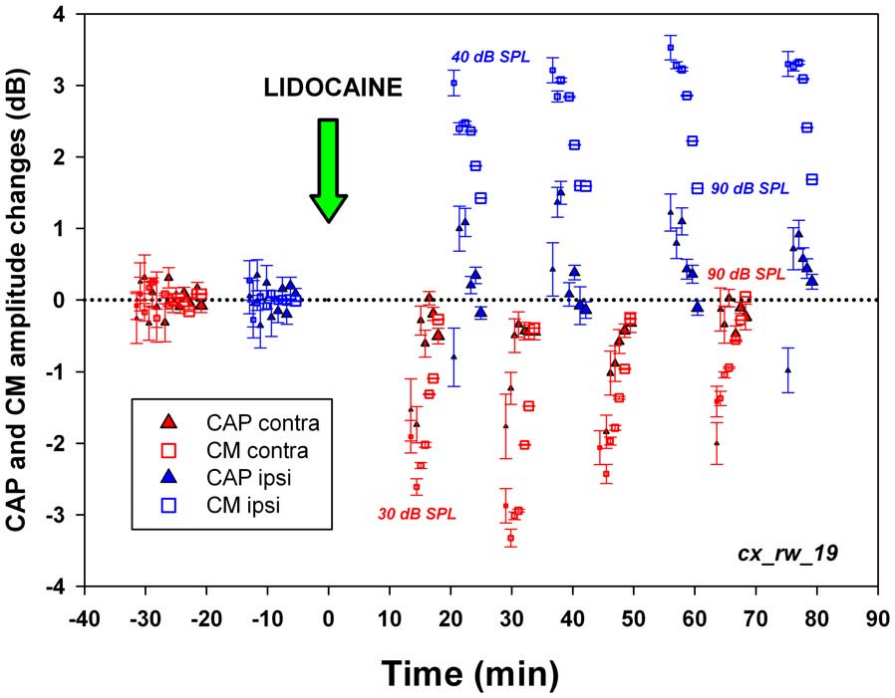
Temporal course and sound pressure dependence of bilateral cortico-olivocochlear effects after lidocaine cortical deactivation. CM (squares) and CAP (triangles) amplitude changes are depicted in dB. Contralateral (right) and ipsilateral (left) cochlear potentials are depicted in red and blue respectively. In this experiment, 2 kHz stimuli were presented at different sound pressure levels, represented by symbol sizes increasing in 10 dB steps from 30–40 to 90 dB SPL. Significant contralateral reductions in CAP and CM (t = 4.20, p<0.01 and t = 6.47, p<0.01 respectively) and significant ipsilateral increases in CAP and CM (t = −2.21, p<0.05 and t = −12.64, p<0.001 respectively) were obtained. The largest effects on CM and CAP were found with low intensity sounds. Note that the sound pressure dependence of amplitude changes in CM and CAP for ipsi and contralateral cochlear electrical responses were in opposite directions (Exp_ID: cx_rw_19).

Lidocaine microinjections produced CAP and CM amplitude changes in all chinchillas studied (n = 10, data shown in Figures 3, 4, 5, 6 and S3). Although the most common effects were CM and CAP amplitude reductions (seven experiments each), we observed a variety of cortico-olivocochlear effects (including both reductions and augmentations in CAP and CM amplitudes) produced by the microinjections (Table 1). Significant CM reductions were commonly accompanied by CAP reductions (n = 6), but in one case of CM decreases, we also obtained CAP enhancements (Figure 3). The means of the maximum CAP and CM lidocaine reductions were −4.3±2.5 dB and −3.4±2.0 dB (mean ± SD) respectively, while the means of the maximum CAP and CM increases were 4.5±0.8 dB and 2.7±0.6 dB, respectively.

In four lidocaine experiments cochlear modulations were evaluated at several frequencies (1 to 8 kHz) and sound pressure levels (20–100 dB SPL) (Figure 4). We describe two types of effects; the first corresponds to CAP and CM reductions, which are evident in the 2–4 kHz frequency band. The second type of effect is restricted to specific frequencies and involves CAP and CM increases for tones <2 kHz and ≥4 kHz, while most other frequencies were not affected (green color) by the pharmacological deactivation.

An example of the temporal course of the cortico-olivocochlear effect produced by a lidocaine microinjection is shown in Figure 5. Sixty minutes after the pharmacological deactivation, ACEP amplitudes were completely recovered; however only a partial recovery was observed in cochlear responses. Note that CM and CAP enhancements were larger for low to moderate sound pressure levels than for high intensities. An example of the temporal course of bilateral and simultaneous cortico-olivocochlear effects is displayed in Figure 6. In all cases we obtained larger CAP and CM increases for low sound pressure levels (four out of four cochleae); however greater CAP and CM reductions with low intensity stimuli were observed in 75% of the cases (six out of eight cochleae).

### Cryoloop deactivation experiments (n = 5)

**Figure 7:**
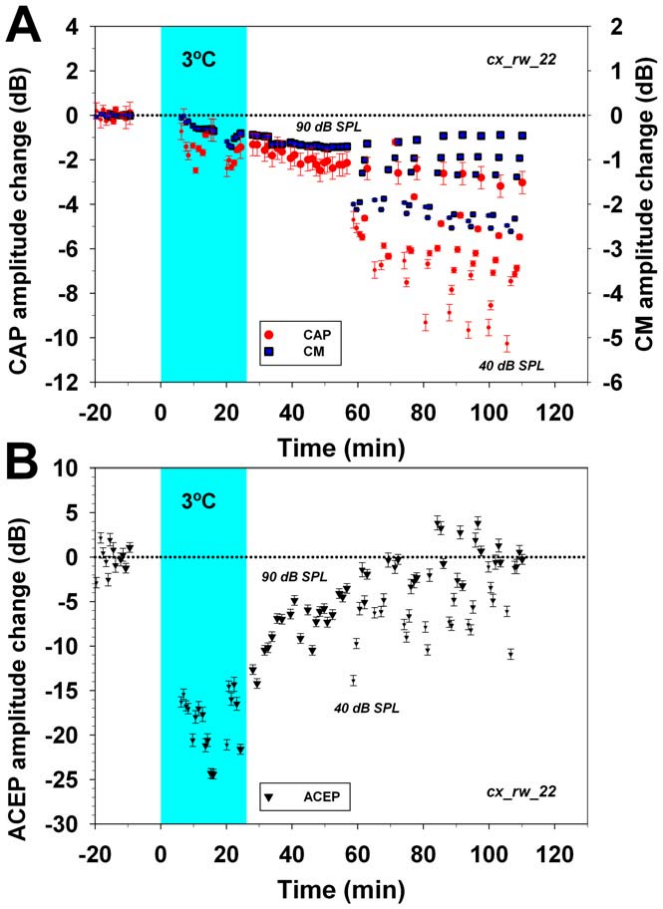
Temporal course of CAP, CM and ACEP changes before, during and after auditory cortex cooling at 3°C with cryoloops. Amplitude changes in CM (blue squares), CAP (red circles) and ACEP (black triangles) are depicted in dB referenced to the mean baseline amplitude prior cortical cooling. In this experiment, 2 kHz stimuli were presented at different sound pressure levels, represented by symbols sizes increasing in 10 dB steps from 40 to 90 dB SPL. Cyan shaded areas illustrate the period of cortical cryoloop cooling at 3°C. **A. Amplitude changes in cochlear potentials.** Auditory cortex cooling produced significant CAP and CM amplitude reductions (F = 50.42, p<0.001 and F = 132.66, p<0.001 respectively), which were largest for low and moderate sound pressure levels. Tukey post-hoc tests revealed significant CAP and CM amplitude differences between the three periods studied (baseline, cooling and recovery). **B. Auditory-cortex evoked potentials amplitude changes.** Cortical cooling reduced ACEP amplitudes down to −25 dB. Note that 40 to 60 minutes after the end of cortical cooling there was a complete recovery of evoked potentials, but there was no recovery in cochlear potentials amplitudes.

**Figure 8:**
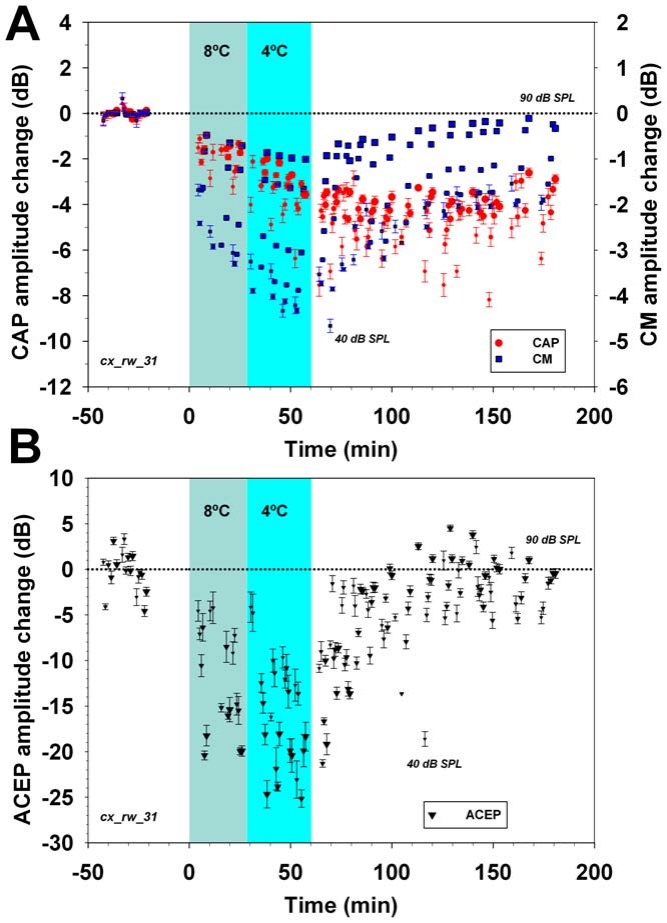
Temporal course of amplitude changes in CAP, CM and ACEP before, during and after auditory cortex cooling at 8° and 4°C with cryoloops. In this experiment 3 kHz stimuli were presented at different sound pressure levels, represented by symbols sizes increasing in 10 dB steps from 40 to 90 dB SPL. Light blue and cyan shaded areas illustrate periods of cortical cooling at 8° and 4°C respectively. **A. Cochlear potentials amplitude changes.** Note that there is a significant reduction in CAP and CM amplitudes (F = 113.74, p<0.001 and F = 40.55, p<0.001 respectively) at 8° and 4°C. Tukey post-hoc test revealed CAP amplitude differences between the four periods studied (baseline, cooling at 8°, at 4°, and recovery), but, in the case of CM amplitude changes, only for baseline against all other periods. After turning off the cryoloop pump, there was a recovery of CM for responses obtained at 80 and 90 dB SPL. In all other cases, the amplitudes of cochlear responses remained reduced even two hours after the cerebral blood flow restored the normal auditory cortex temperature (passive warming). **B. ACEP amplitude changes.** Note that auditory cortex cooling at 8°C was sufficient to reduce auditory-cortex potentials. Cooling at 4°C produced a greater decrease in cortical evoked potentials, and fifty to sixty minutes after the end of cortical cooling there was a complete recovery of ACEP amplitudes.

Auditory cortex cooling with cryoloops (2° to 8°C) produced reversible amplitude reductions in cortical evoked potentials in all experiments (n = 5, data shown in Figures 7, 8, and S4). Significant CAP and CM reductions were obtained by cooling the auditory cortex to 2–4°C, but also at 8°C. The means of the maximum CAP and CM cryoloop reductions were −5.0±2.4 dB and −2.7±1.1 dB respectively. Similar to the lidocaine experiments, the magnitudes of the cortico-olivocochlear effects were dependent on sound pressure levels and in two-thirds of the cases were greatest for low to moderate sound pressure levels (Figures 7 and 8).

After turning off the cryoloop cold methanol pump, there was a passive restoration of auditory cortex temperature. A complete recovery in the amplitude of cortical evoked potentials was obtained during this period of passive warming (Figures 7, 8 and S4). On the other hand, a reversible cochlear effect was only observed with high intensity stimuli (80 and 90 dB SPL), while a sustained cortico-olivocochlear efferent effect was observed at low to moderate sound pressure levels (Figure 8).

To rule out the possibility of cochlear cooling produced by the cortical cryoloop, in two experiments we positioned a second thermocouple in the promontory of the cochlea and simultaneously measured cochlear and auditory cortex temperatures. Although the cortical surface was cooled to 2°C, cochlear temperature changes were <1°C (Figure S5), meaning that amplitude modulations of cochlear potentials produced by cortical cryoloops were not produced by direct cooling of the cochlea.

**Figure 9:**
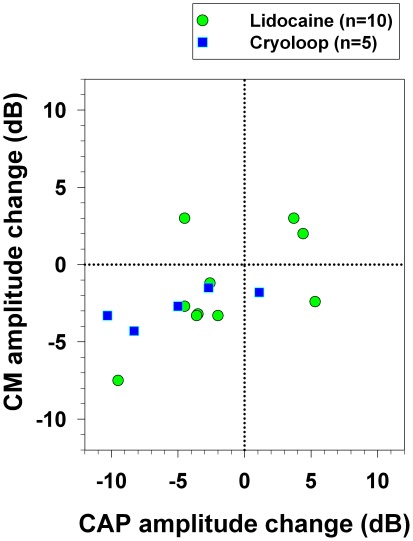
Summary of contralateral corticofugal effects obtained with lidocaine and cryoloop deactivation experiments. A scatter plot of maximum CAP and CM amplitude changes obtained with both deactivation methods is shown (green circles: lidocaine; blue squares: cryoloops). The most common effect was a simultaneous reduction in CM and CAP, observed in ten experiments. Parallel effects (correlative increases or decreases) were obtained in twelve animals, while dissociated effects were seen in three. Note that lidocaine deactivation produced diverse types of CAP and CM amplitude changes (reductions and increases), while most of the cryoloop experiments produced significant CM and CAP reductions.

A summary of the increases and decreases of CAP and CM amplitudes obtained in contralateral deactivation experiments with lidocaine and cryoloops are displayed in Figure 9. Note that the most common cortico-olivocochlear efferent effect in contralateral ear was a concomitant decrease in CAP and CM (n = 10), and that CM significant increases were only achieved in lidocaine experiments. Dissociated effects (an increase in one type of cochlear potential simultaneous to a decrease in the other) were only observed in three animals, while in twelve animals we obtained parallel amplitude changes (including correlative CAP and CM reductions or increases). Moreover, if we include ipsi and contralateral experiments (a total of 17 ears), fourteen cochleae had correlative changes in CAP and CM (Table 1).

### Lesion experiments (n = 5)

**Figure 10:**
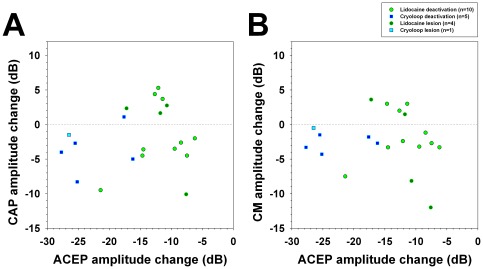
CAP and CM amplitude changes in function of cortical deactivation measured by ACEP attenuation. Data from deactivation and lesion experiments were included in this figure (n = 20). **A.** Scatter plot of maximum ACEP and CAP amplitude changes. **B.** Scatter plot of maximum ACEP and CM amplitude changes. Note that CAP and CM augmentations were obtained in the same range of ACEP reductions between −10 to −20 dB, and that the cryoloop technique produced larger decreases in ACEP amplitude than lidocaine microinjections.

The mean largest ACEP amplitude reduction in the lidocaine lesion group was −11.8±2.3 dB, while in the cryoloop alone lesion experiment it was −26.5 dB. Auditory cortex lesion also produced CAP and CM amplitude changes (Table 1) that were sustained in time after the cortical lesion. Scatter plots of maximum CAP and CM amplitude changes in function of ACEP reductions are presented in Figure 10, including both deactivation and lesion experiments. CAP and CM augmentations were only observed with ACEP reductions over a range between −10 to −20 dB, while ACEP reductions larger than 20 dB yielded only CM and CAP reductions.

## Discussion

Both types of auditory cortex deactivation and lesion experiments produced changes in the amplitudes of cochlear potentials. These findings demonstrate that basal activity in the auditory cortex regulates cochlear activity and afferent responses, probably through cortico-olivocochlear pathways. In agreement with previous work, the most common efferent effect during cortical deactivation was a reduction in the amplitude of CM responses [23], [24]. In the present work, we simultaneously recorded CAP and CM responses, which allowed us to observe a variety of other effects at the receptor and auditory-nerve levels, obtaining evidence that the physiology of the cortico-olivocochlear system is more complex than has been described previously.

### Two parallel descending pathways from the auditory cortex to the cochlea

We demonstrated that amplitude changes in CM can occur together with either CAP increases or reductions, suggesting the presence of two descending functional pathways from cortex to cochlea. The olivocochlear system has two anatomically and functionally different sub-systems: the lateral and medial olivocochlear fibers [8], [36]. Electrical activation of MOC fibers decreases CAP amplitudes and increases CM potentials [14], [15], while LOC activity can reduce or increase CAP amplitudes, without affecting CM potentials [16]–[18]. Electrophysiological studies at the level of the inferior colliculus suggest the presence of two functionally different colliculo-cochlear pathways which modulate the activity of medial and lateral olivocochlear fibers, thus regulating that of outer hair cells, and of auditory nerve afferent fibers respectively [19], [20], [29], [30].

As we found CM amplitude changes in all experiments, and due to the fact that the MOC system is needed to modulate CM amplitudes [36], we assume that MOC neurons were always affected by cortical deactivations. In addition, most CAP and CM reductions were obtained with stimulus frequencies between 2–4 kHz (see Figure 4) while in the majority of the experiments, the largest effects were found using the lowest level stimuli (see Figures 5, 6, 7 and 8). These cortico-olivocochlear frequency and intensity dependencies are similar to those obtained during electrical stimulation of MOC fibers in the chinchilla [15], clearly suggesting a modulation of MOC activity produced by auditory-cortex deactivation.

**Figure 11:**
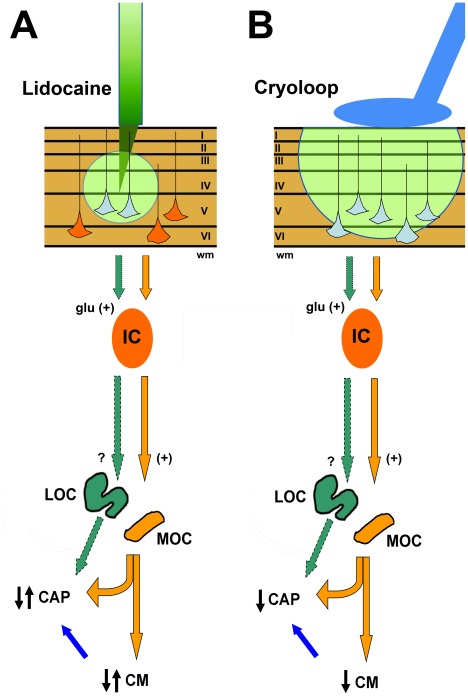
Schematic representation of the working model for corticofugal modulation of cochlear and auditory nerve responses produced by auditory cortex deactivation. The magnitude of cortical deactivation (green shade) produced by cryoloops (B) was larger than the one produced by lidocaine microinjections (A). Based on previous studies [48]–[50] we propose the presence of an excitatory basal tone mediated by two groups of glutamatergic pyramidal neurons. In this model, pyramidal neurons located in auditory cortex layers V and VI project through parallel pathways to the inferior colliculus (IC). These cortical neurons are differentially deactivated (represented in light-blue) by lidocaine or cryoloops. There is indirect evidence of excitatory connections from the IC to MOC neurons [52], but it is unknown whether there is another parallel pathway from IC to the LOC (? mark in the model). In this working model, a complete loss of descending excitatory activity will lead to CAP and CM reduction, as was observed in most cryoloop experiments. On the other hand, random deactivation of different subsets of pyramidal neurons could lead to CAP and CM enhancements or reductions, depending on the population of neurons deactivated. Blue arrows from CM to CAP represent an alternative hypothesis to explain CAP changes. Corticofugal modulation of outer hair cell activity can alter cochlear functioning and thus auditory nerve responses. (I–VI: auditory cortex layers; wm: white matter; glu: glutamate; IC: inferior colliculus).

According to evidence obtained with MOC electrical stimulation in anesthetized animals, increasing or reducing MOC activity will produce dissociated CAP and CM amplitude changes [14], [15]. If during auditory cortex deactivation there was a selective increase or reduction of MOC activity, CAP reductions and CM increases, or an inverse pattern (CAP increases and CM reductions) should be expected. However, dissociated effects were observed in only three deactivation experiments. On the other hand, CM and CAP parallel amplitude changes obtained in twelve deactivation experiments were suggestive of a simultaneous modulation of MOC and LOC activity (See Figure 11 for a working model of cortico-olivocochlear modulations). Another possibilities that could partially explain the diversity of the effects obtained are the intrinsic variability of MOC activity between different subjects [37], [38], or differences in ketamine anesthesia level [39]–[41].

### Basal efferent tone

Our results demonstrate that blocking spontaneous auditory cortex activity modulates CM and CAP responses, suggesting that cortical descending pathways in the auditory system regulate cochlear ipsi and contralateral responses through a basal efferent tone.

There is previous evidence that the MOC and LOC systems can either increase or decrease cochlear sensitivity through a basal tone. Zheng and collaborators [31] found CM amplitude reductions concomitant to increases in the amplitude of distortion product otoacoustic emissions in chronically de-efferented chinchillas in which MOC fibers at the midline of the fourth ventricle floor were sectioned. In addition, cochlear perfusion with a dopamine antagonist modulated auditory-nerve activity in guinea pigs, suggesting the presence of a basal tone in the LOC system [16]. Taken together, these experiments strongly suggest the presence of a basal tone in the olivocochlear system (MOC and LOC) that regulates cochlear afferent responses.

The present results demonstrate that under basal conditions the auditory cortex exerts a modulation of the cochlear and auditory nerve afferent responses through efferent pathways, implying the presence of a cortico-olivocochlear tone that increases or decreases cochlear sensitivity. The interaction between the corticofugal and olivocochlear efferent basal tones remains elusive. We propose an excitatory modulation of the MOC and LOC systems by two descending pathways from the auditory cortex (Figure 11).

### Sustained cochlear effect after cortical deactivation

A sustained cochlear effect, larger for weak than for strong stimuli, was observed in most experiments (see Figures 5, 6, 7, 8, S3 and S4), even after complete restoration of ACEP amplitude. A possible explanation for this phenomenon is the fact that auditory-cortex afferent and efferent activity depend on different neural populations.

While disruption of the efferent tone was probably attained by inactivating pyramidal neurons located at cortical layers V and VI, ACEP amplitude relies mostly on afferent activity from neurons located at cortical layer IV. One hour after auditory cortex deactivation, afferent activity from cortical layer IV is recovered, as reflected by the amplitude of auditory-cortex evoked potentials, however the cortical dynamics maintaining the efferent tone to the cochlea are not. This idea is supported by the fact that although sixty minutes after cortical deactivation there is a recovery in the amplitude of cortical evoked potentials, other properties of the response, such as latency and waveform, are still disrupted as compared to baseline evoked potentials (Figure S6), thus suggesting a sustained alteration in cortical function.

### Cortical deactivation techniques

Similar to previous reports, the time course of the lidocaine effect produced a reversible deactivation of cortical evoked potentials from 30 to 90 minutes [32], [42]. According to Tehovnik and Sommer [32], a 3 µl lidocaine microinjection is expected to spread about one millimeter in radius, and thus deactivate a cortical area that comprises only the primary auditory cortex of the chinchilla [34], [35]. In the present experiments, lidocaine microinjections were useful for transiently deactivating the primary auditory cortex, which is the main origin of descending projections to the cochlea [43].

Discrete cortical areas can be deactivated with cryoloops [33], [44]. Although the technique was initially described in cats, recent publications demonstrated that it could be used in the auditory cortex of rodents with no cooling of subcortical structures [45]–[47]. Moreover, in the present experiments, no significant changes (<1°C) were found in cochlear temperature during cortical cooling up to 2°C (Figure S5). For these reasons we rule out any possibility of cochlear cooling produced by the cryoloop, and thus the cochlear effect can be attributed to the efferent system.

Remarkably, the evidence that cochlear responses are modulated by auditory cortex basal activity is supported by similar findings obtained with two independent deactivation techniques. Although the corticofugal effects were comparable in magnitude for the lidocaine and cryoloop experiments, one difference was that with the latter method, mostly significant CAP and CM reductions were found (Figure 9). This fact could be attributed to differential thresholds of cortical deactivation between these two techniques, implying that a subset of pyramidal neurons is more sensitive to lidocaine microinjections. Another hypothesis to explain this difference could be the size of the cryoloops, as the extension of the cortical deactivated area is larger with a cryoloop (3–4 mm) than after a lidocaine microinjection (1 mm). Similarly, average ACEP reductions were larger with cryoloops than with lidocaine (−22.4 dB versus −11.3 dB). We consider that an extended area of deactivation affects all descending pathways, including those that modulate MOC and LOC neurons. That type of deactivation should produce correlative changes in cochlear potentials amplitudes, as it was actually obtained in most cryoloops experiments.

A proposed model for corticofugal modulation of cochlear electrical responses is presented separately for lidocaine and cryoloop experiments in Figure 11. There is evidence that auditory cortex pyramidal neurons from layer V which project to the inferior colliculus are glutamatergic [48]. Moreover, anatomic and physiological evidence demonstrates the presence of two different populations of layer V neurons [49], [50]. In our working model we propose that these two cellular groups project through parallel pathways to the inferior colliculus. The presence of colliculo-olivocochlear pathways has been anatomically demonstrated [51], and functional experiments suggest an excitatory transmission from IC to MOC [19], [52]; however the existence of IC connections to the LOC still remains elusive.

### Possible functions and clinical role of cortico-olivocochlear pathways

Here, we demonstrate that auditory cortex basal activity modulates cochlear responses in anesthetized chinchillas, and that the main cortico-olivocochlear effect was a parallel reduction in cochlear potentials. Neuroanatomical evidence shows that the corticofugal descending auditory system comprises several feedback loops: (i) the olivocochlear system, (ii) colliculo-cochlear pathways, and (iii) cortico-olivocochlear descending projections [1], [6], [7], [53]. Auditory efferent functions can be classified according to the neural circuit most probably involved. Efferent functions possibly depending mainly on the olivocochlear acoustic reflex which comprises brainstem loops [54], are protection to acoustic trauma [55] and balance of interaural cochlear sensitivity [56]. Efferent functions that probably involve cortico-olivocochlear pathways, include control of auditory afferent responses during sleep stages [57] and selective attention to auditory or visual stimuli [58], [59].

Parallel amplitude changes in CAP and CM have been reported during sleep stages in guinea pigs, which probably involve cortical modulation of MOC and LOC activities [57]. In contrast, dissociated changes in the amplitudes of cochlear potentials have been found during selective attention to visual stimuli [59], suggesting in this case a selective modulation of MOC activity by the auditory cortex. In the present work we found only three deactivation experiments suggestive of selective cortical modulation of MOC activity (see Table 1). Increasing the spatial precision of cortical deactivation/stimulation techniques will allow future research to selectively affect regions that modulate MOC or LOC activity.

Cortical modulation of cochlear responses produced during auditory cortex deactivation could be a possible mechanism involved in tinnitus suppression. MOC function, as assessed by contralateral acoustic stimulation, is reduced in patients with normal hearing and tinnitus [60]. In addition, auditory cortex electrical stimulation suppresses tinnitus in humans and rats [61], [62]. However, whether the suppressive effect is produced by cortico-olivocochlear efferent activation or by cortical induced plasticity remains elusive [63].

### Conclusions

We have demonstrated that deactivation of auditory cortex basal activity modulates the amplitude of cochlear and auditory nerve responses in chinchillas. Our results show that there is a modulation of the olivocochlear system by the cerebral cortex. Furthermore, the diversity of the observed effects suggests the presence of at least two functional pathways from the auditory cortex to the cochlear receptor.

## Supporting Information

Figure S1 **Experimental paradigms.** **A. Lidocaine experiments.** Two different time periods were evaluated in this set of experiments: before (white period) and after (green period) lidocaine microinjection at time “0 min”. CAP, CM and ACEP input-output curves using sequential stimuli ranging from 20 to 100 dB SPL were recorded during these two periods comprising from 40 minutes before and to 130 minutes after the lidocaine microinjection. **B. Cryoloop experiments.** CAP, CM and ACEP input-output curves using sequential stimuli from 30 to 90 dB SPL were recorded over three or four periods: baseline, cooling (at one or two temperatures) and recovery. Baseline amplitudes were evaluated from −50 minutes up until the beginning of cortical cooling (Time 0). The duration of the cortical cooling period (light blue shading) was from 20 to 60 minutes. In two cryoloop deactivation experiments (cx_rw_31 and cx_rw_23) there was a second cooling period (blue shading). The recovery period begins at the end of cortical cooling.
(TIF)

Figure S2 **Example of ACEP before and after a saline microinjection (3 µl at a rate of 1 µl/min).** Although, no significant ACEP amplitude changes were obtained, there was an increase in variability after saline microinjections (Exp_ID: cx_rw_14).
(TIF)

Figure S3 **Examples of CAP, CM and ACEP amplitudes changes after cortical lidocaine microinjection.** **A. Experiment cx_rw_01.** Significant CAP and CM reductions (t = 7.99, p<0.001 and t = 11.27, p<0.001 respectively) were noted after cortical microinjection with lidocaine. Note that 60 minutes after the lidocaine microinjection there is a rebound in the amplitude of ACEP but a sustained reduction in CAP and CM amplitudes. **B. Experiment cx_rw_13.** Significant reductions in CAP and CM (t = 9.29, p<0.001 and t = 9.98, p<0.001 respectively) after cortical microinjection with lidocaine.
(TIF)

Figure S4 **Examples of CAP, CM and ACEP amplitudes changes during auditory cortex deactivation using cryoloops.** Panels A,B and C show CAP and CM changes, while panels D, E and F show ACEP amplitude changes. Each column correspond to one experiment. **A and D, experiment cx_rw_23:** Auditory cortex cooling produced significant CAP increases and CM reductions (F = 13.85, p<0.001 and F = 209.62, p<0.001 respectively), which were the largest at high sound pressure levels. Tukey post-hoc tests revealed CM amplitude differences between the cooling (at 4° and 2°C) and recovery periods compared with the baseline period; while the only significant difference in CAP was between cooling at 2°C and the baseline period. **B and E, experiment cx_rw_30:** Auditory cortex cooling produced significant reductions in CAP and CM (F = 53.99, p<0.001 and F = 26.89, p<0.001 respectively) at low and moderate sound pressure levels. Tukey post-hoc tests revealed CAP and CM amplitude differences between baseline periods against cooling at 8°C and the recovery period, but no difference between the cooling and recovery periods. **C and F, experiment cx_rw_17:** Auditory cortex cooling produced significant CAP and CM amplitude reductions (F = 98.66, p<0.001 and F = 149.60, p<0.001 respectively). Tukey post-hoc tests revealed CAP and CM amplitude differences between baseline periods against cooling at 2°C and the recovery period, but no difference between the cooling and recovery periods.
(TIF)

Figure S5 **No significant cochlear temperature changes (<1 C°) were noted during cortical cooling using a cryoloop at 8° and 4°C.** There is no relation between sound level and temperature, but as data was acquired with sequential input-output curves, this graph shows temperature stability in recordings obtained before, during and after cortical cooling (Exp_ID: cx_rw_31).
(TIF)

Figure S6 **ACEP waveforms before, during and after cooling at 8°C with cryoloops.** Note a recovery, but a delay in latency and widening of the averaged ACEP waveforms during the recovery period. ACEP waveforms were recorded 30 minutes before the beginning of the cooling period, during the cooling period, and 50 minutes after the end of the cooling period (Exp_ID: cx_rw_30).
(TIF)
