# Supplementary figures and images for: Auditory Cortex Basal Activity Modulates Cochlear Responses in Chinchillas
Source: PLoS One. 2012 Apr 30;7(4):e36203. doi: 10.1371/journal.pone.0036203 (PMC3340362; doi:10.1371/journal.pone.0036203)

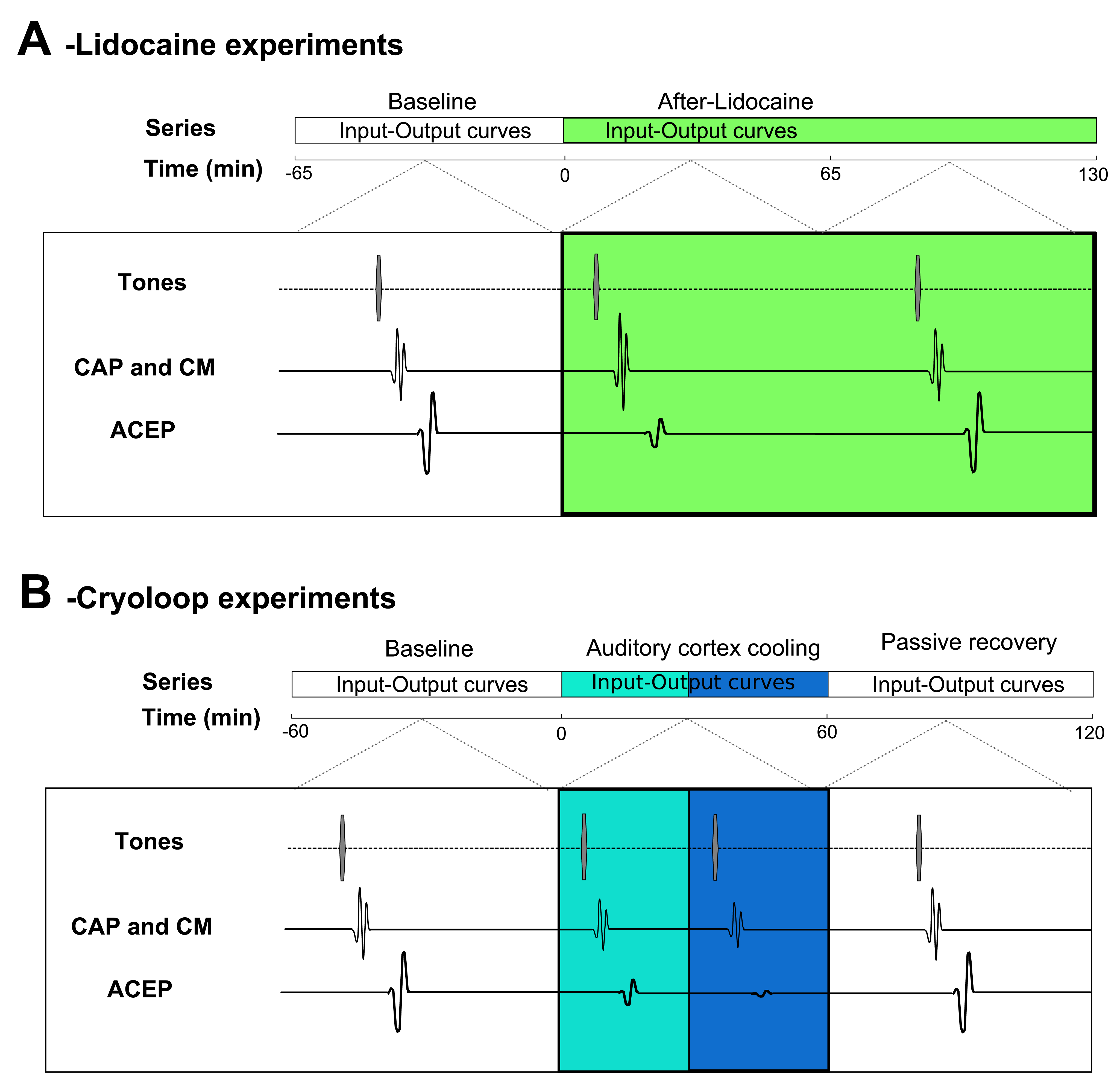

Supplement: Figure S1 — Experimental paradigms. A. Lidocaine experiments. Two different time periods were evaluated in this set of experiments: before (white period) and after (green period) lidocaine microinjection at time “0 min”. CAP, CM and ACEP input-output curves using sequential stimuli ranging from 20 to 100 dB SPL were recorded during these two periods comprising from 40 minutes before and to 130 minutes after the lidocaine microinjection. B. Cryoloop experiments. CAP, CM and ACEP input-output curves using sequential stimuli from 30 to 90 dB SPL were recorded over three or four periods: baseline, cooling (at one or two temperatures) and recovery. Baseline amplitudes were evaluated from −50 minutes up until the beginning of cortical cooling (Time 0). The duration of the cortical cooling period (light blue shading) was from 20 to 60 minutes. In two cryoloop deactivation experiments (cx_rw_31 and cx_rw_23) there was a second cooling period (blue shading). The recovery period begins at the end of cortical cooling. (TIF) [file pone.0036203.s001.tif]

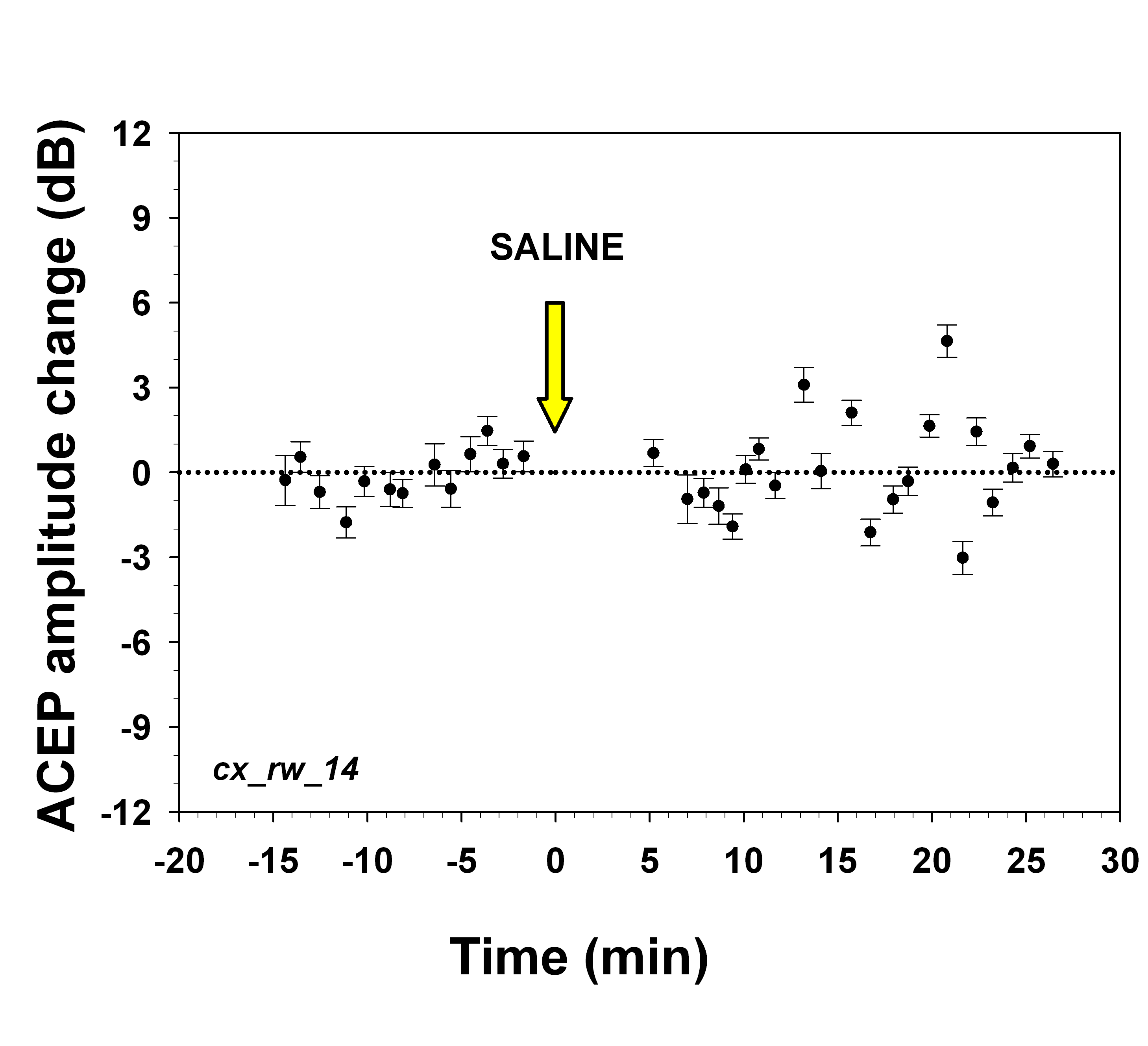

Supplement: Figure S2 — Example of ACEP before and after a saline microinjection (3 µl at a rate of 1 µl/min). Although, no significant ACEP amplitude changes were obtained, there was an increase in variability after saline microinjections (Exp_ID: cx_rw_14). (TIF) [file pone.0036203.s002.tif]

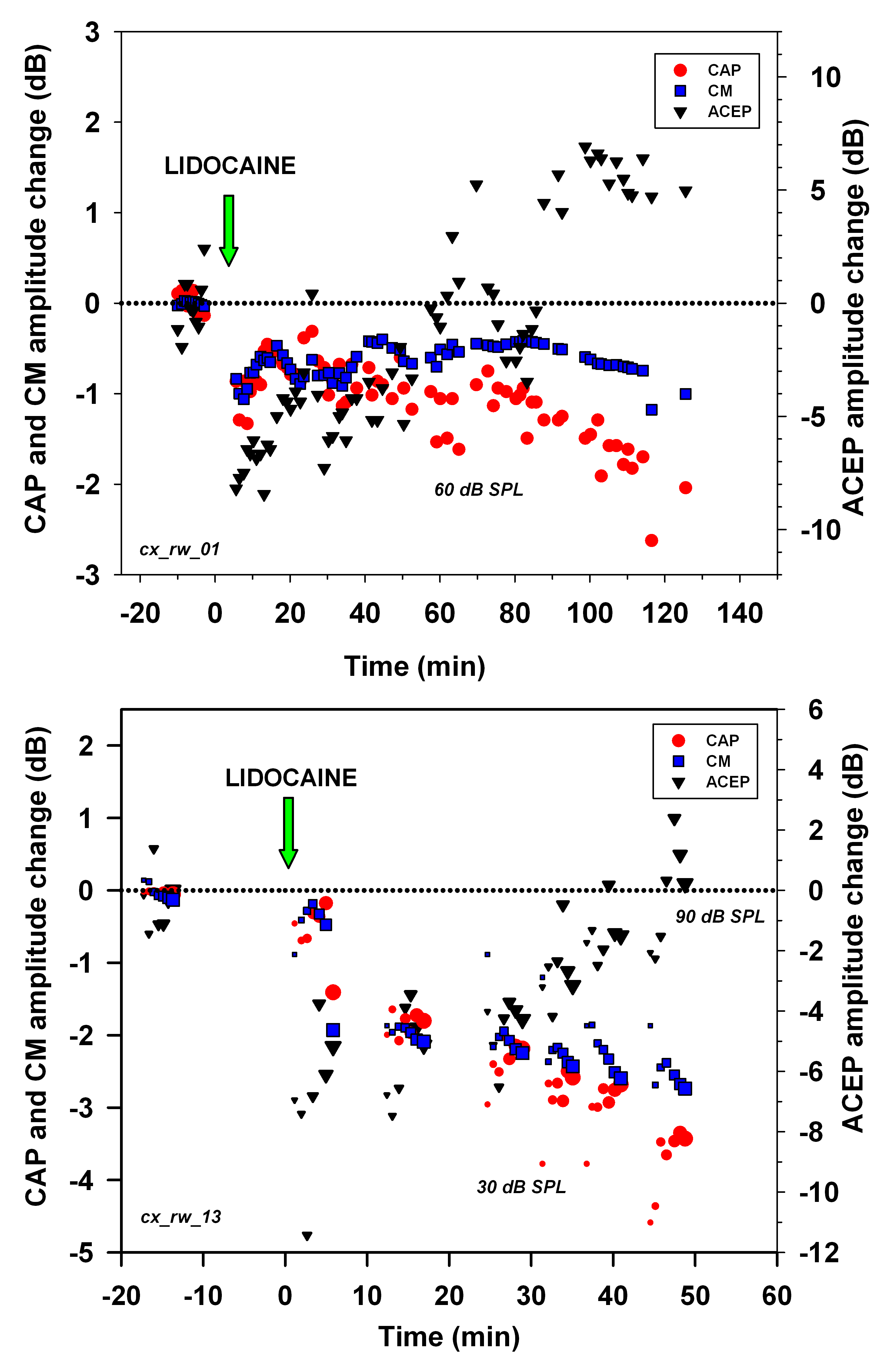

Supplement: Figure S3 — Examples of CAP, CM and ACEP amplitudes changes after cortical lidocaine microinjection. A. Experiment cx_rw_01. Significant CAP and CM reductions (t = 7.99, p<0.001 and t = 11.27, p<0.001 respectively) were noted after cortical microinjection with lidocaine. Note that 60 minutes after the lidocaine microinjection there is a rebound in the amplitude of ACEP but a sustained reduction in CAP and CM amplitudes. B. Experiment cx_rw_13. Significant reductions in CAP and CM (t = 9.29, p<0.001 and t = 9.98, p<0.001 respectively) after cortical microinjection with lidocaine. (TIF) [file pone.0036203.s003.tif]

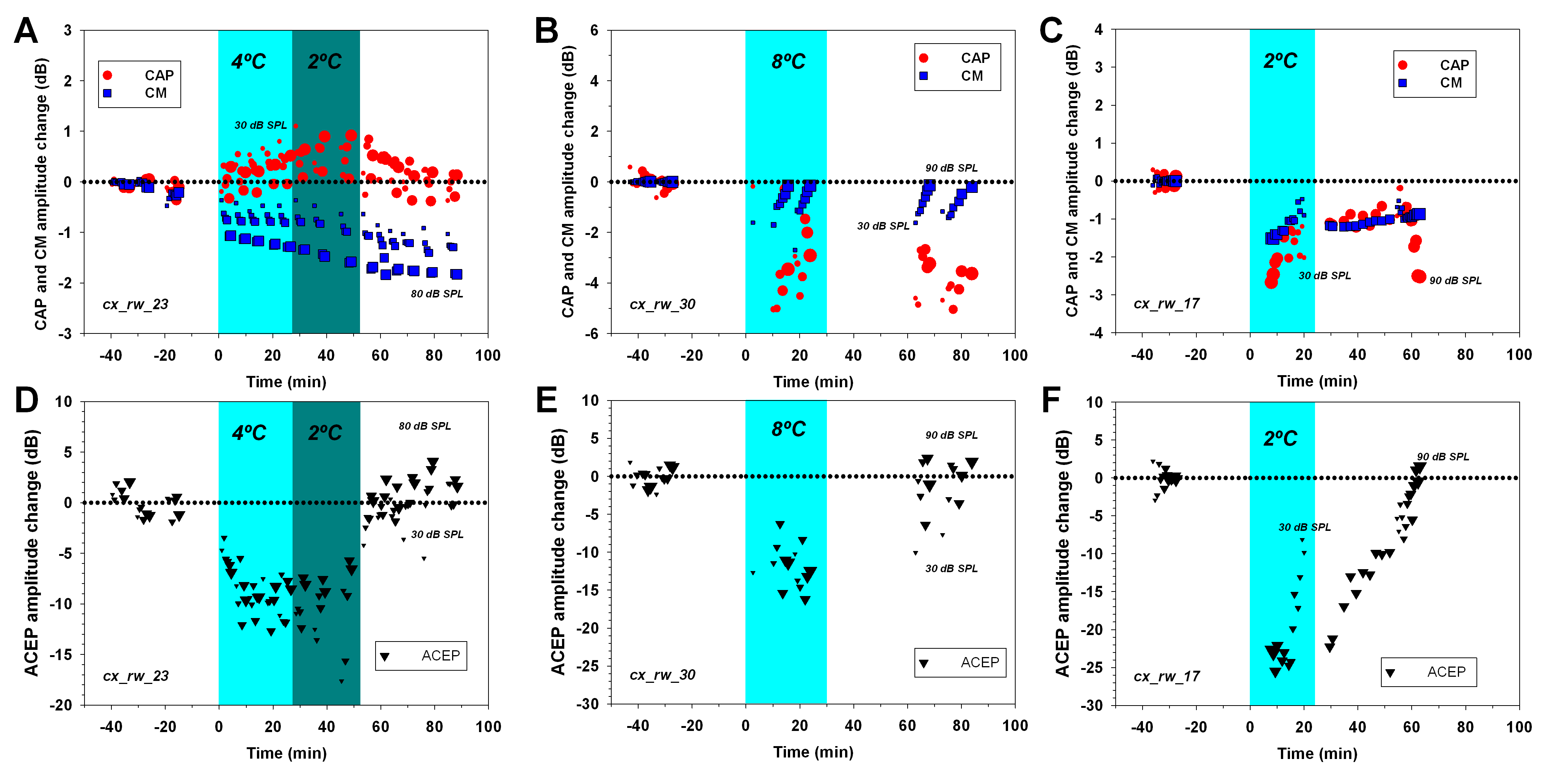

Supplement: Figure S4 — Examples of CAP, CM and ACEP amplitudes changes during auditory cortex deactivation using cryoloops. Panels A,B and C show CAP and CM changes, while panels D, E and F show ACEP amplitude changes. Each column correspond to one experiment. A and D, experiment cx_rw_23: Auditory cortex cooling produced significant CAP increases and CM reductions (F = 13.85, p<0.001 and F = 209.62, p<0.001 respectively), which were the largest at high sound pressure levels. Tukey post-hoc tests revealed CM amplitude differences between the cooling (at 4° and 2°C) and recovery periods compared with the baseline period; while the only significant difference in CAP was between cooling at 2°C and the baseline period. B and E, experiment cx_rw_30: Auditory cortex cooling produced significant reductions in CAP and CM (F = 53.99, p<0.001 and F = 26.89, p<0.001 respectively) at low and moderate sound pressure levels. Tukey post-hoc tests revealed CAP and CM amplitude differences between baseline periods against cooling at 8°C and the recovery period, but no difference between the cooling and recovery periods. C and F, experiment cx_rw_17: Auditory cortex cooling produced significant CAP and CM amplitude reductions (F = 98.66, p<0.001 and F = 149.60, p<0.001 respectively). Tukey post-hoc tests revealed CAP and CM amplitude differences between baseline periods against cooling at 2°C and the recovery period, but no difference between the cooling and recovery periods. (TIF) [file pone.0036203.s004.tif]

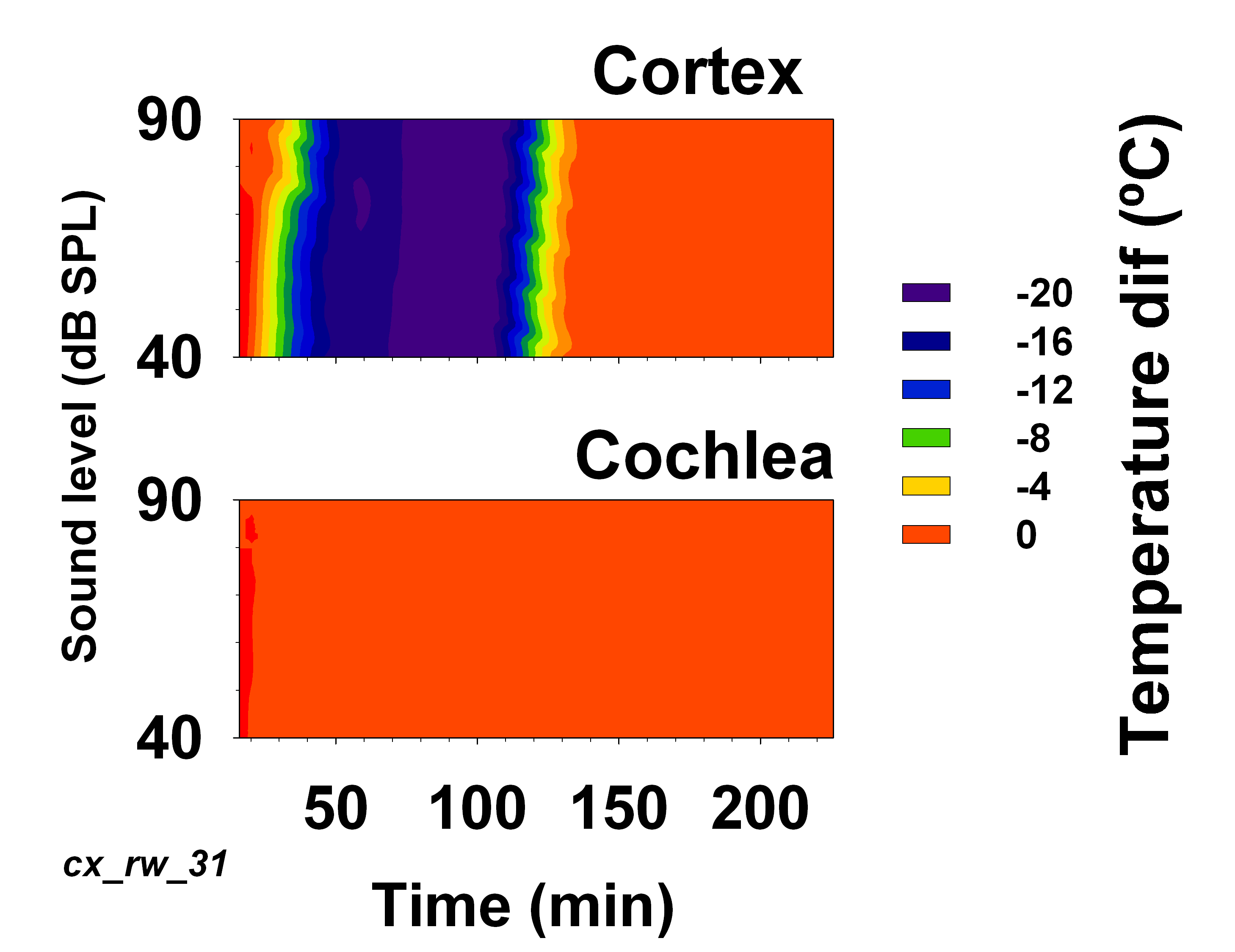

Supplement: Figure S5 — No significant cochlear temperature changes (<1 C°) were noted during cortical cooling using a cryoloop at 8° and 4°C. There is no relation between sound level and temperature, but as data was acquired with sequential input-output curves, this graph shows temperature stability in recordings obtained before, during and after cortical cooling (Exp_ID: cx_rw_31). (TIF) [file pone.0036203.s005.tif]

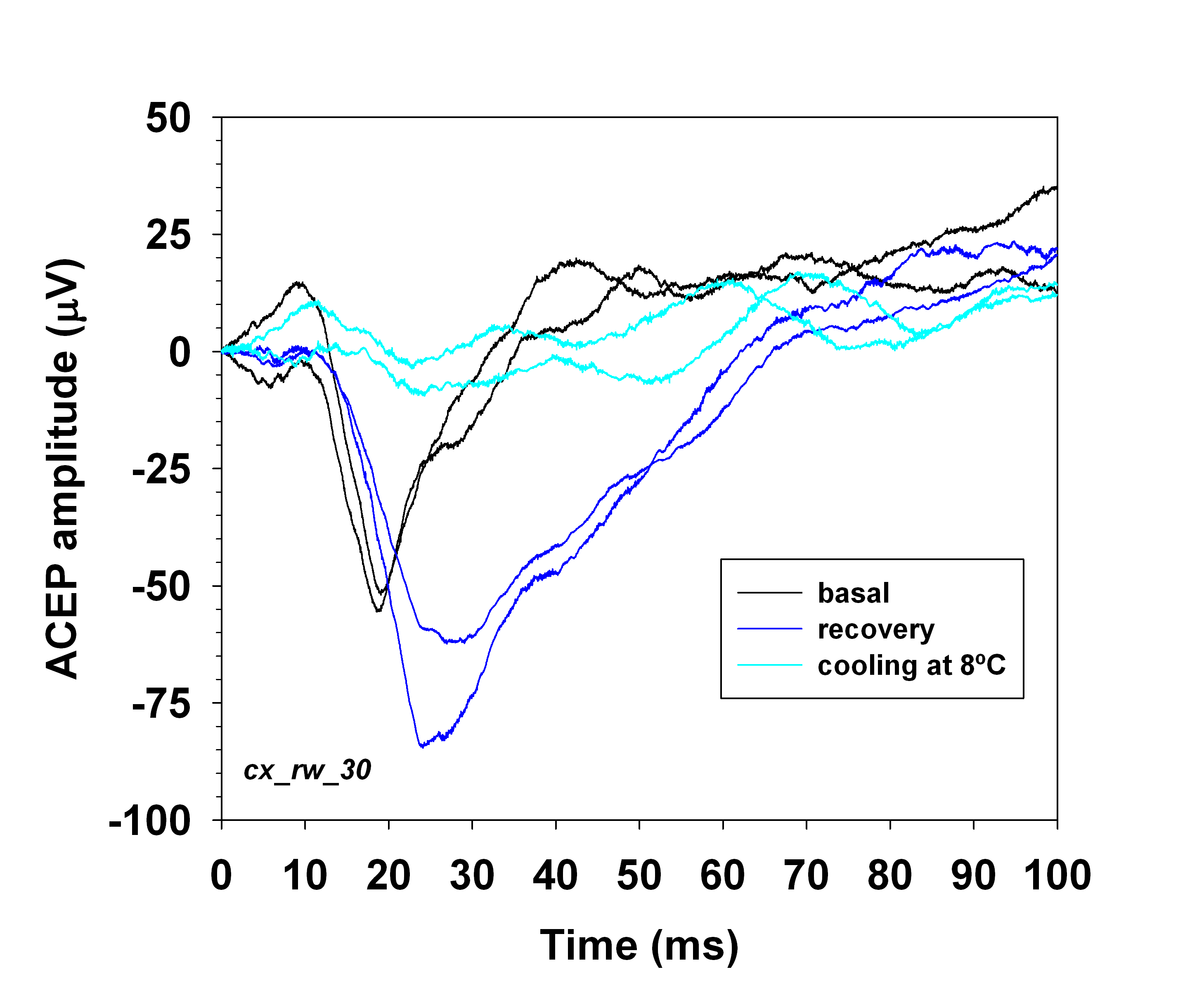

Supplement: Figure S6 — ACEP waveforms before, during and after cooling at 8°C with cryoloops. Note a recovery, but a delay in latency and widening of the averaged ACEP waveforms during the recovery period. ACEP waveforms were recorded 30 minutes before the beginning of the cooling period, during the cooling period, and 50 minutes after the end of the cooling period (Exp_ID: cx_rw_30). (TIF) [file pone.0036203.s006.tif]
